# Designing m-Health interventions for precision mental health support

**DOI:** 10.1038/s41398-020-00895-2

**Published:** 2020-07-07

**Authors:** N. Bidargaddi, G. Schrader, P. Klasnja, J. Licinio, S. Murphy

**Affiliations:** 1 College of Medicine & Public Health, Flinders University, Adelaide, Australia; 2 School of Information, University of Michigan, Ann Arbor, MI USA; 3 Departments of Psychiatry, Pharmacology, Medicine and Neuroscience & Physiology, State University of New York Upstate Medical University, Syracuse, New York USA; 4 Departments of Statistics & Computer Science, Harvard University, Boston, MA USA

**Keywords:** Depression, Psychiatric disorders

## Abstract

Mobile health (m-Health) resources are emerging as a significant tool to overcome mental health support access barriers due to their ability to rapidly reach and provide support to individuals in need of mental health support. m-Health provides an approach to adapt and initiate mental health support at precise moments, when they are most likely to be effective for the individual. However, poor adoption of mental health apps in the real world suggests that new approaches to optimising the quality of m-Health interventions are critically needed in order to realise the potential translational benefits for mental health support. The micro-randomised trial is an experimental approach for optimising and adapting m-Health resources. This trial design provides data to construct and optimise m-Health interventions. The data can be used to inform when and what type of m-Health interventions should be initiated, and thus serve to integrate interventions into daily routines with precision. Here, we illustrate this approach in a case study, review implementation issues that need to be considered while conducting an MRT, and provide a checklist for mental health m-Health intervention developers.

## Introduction

A quarter of the global population experiences mental disorders, and even though effective treatments exist, the majority of affected people never receive any support^1^. At the same time, under resourced health systems are struggling to respond to the burden of mental disorders in an efficient and effective manner. Against this background, the use of mobile technology (m-Health) to monitor and support mental health has generated significant interest. m-Health is the “medical and public health practice supported by mobile devices, such as mobile phones, patient monitoring devices, personal digital assistants (PDAs), and other wireless devices”^2^. Central to the interest in m-Health is the worldwide growth in smartphone popularity and emergence of low-cost wearables with advanced functionality, better connectivity and granular monitoring of behavioural and physiological parameters. Over 10,000 m-Health resources to monitor and support mental health are available for immediate download and use on Google and Apple app stores. Several of these are endorsed by public health organisations such as the UK’s National Health Service^3,4^. These m-Health resources allow people to monitor and receive mental health support in a way that face‐to‐face/paper‐based methods of assessment have, up until now, not allowed, that is, in a way that is flexible and suits their own unique circumstances. For people in low-income countries and rural regions where resources are scarce, a standalone self-help m-Health resource might be the only option to receive mental health support. On the other hand, for someone receiving treatment from a mental health professional, an m-Health resource can be an avenue to receive mental health support between sessions or help mental health professionals monitor a patient’s progress outside sessions. But the availability, interest and efficacy of m-Health resources alone have not translated into the realisation of the potential benefits in the real world^5,6^.

Poor uptake and subsiding use of m-Health resources available in the real-world setting indicates that m-Health resources suffer from low engagement^7^. The level of engagement with an m-Health intervention needed from an individual to achieve health benefits can be measured both objectively and subjectively^8^. An example of the former is when and how often one performs the desired behaviour, and how well they perform the behaviour. Subjective measures could include experiences and perceptions from performing the behaviour. Regarding a lack of effective engagement, there are several possible explanations. Very often, poor engagement could be due to an issue with the app design, or problem with the intervention content is not suited for the population, or the intervention is delivered is circumstances in which individuals are less responsive^9^. Furthermore, as mental illnesses reduce one’s motivation and energy levels, engagement with any intervention can become even more difficult. Also the circumstances and needs of individuals are constantly changing over time; inadequate adaptation of interventions to the changing preferences, state and needs of individuals over time^10^ could be an additional important factor contributing to the low engagement problem^11^.

Since individuals are in close proximity to their phone most of the time, the smartphone’s passive sensing and real-time assessment capabilities can be used to observe individuals’ changing circumstances over time^12^. Thus m-Health in mental health care should be able to provide optimized adaptation of interventions so to achieve precision support, i.e., support delivered at the precise moments and circumstances in which the intervention is most useful and the person is most likely to be receptive. In contrast to the one-size-fits-all approach, precision support takes individual variability into account^13^. Using sensor and self-report assessments, phones can deliver interventions dynamically tailored to individuals’ circumstances, specifically when individuals are receptive and most likely to action the intervention behaviour. Harnessing and optimising these m-Health capabilities enables the provision of support that is adapted to each individual’s unique time-varying state.

In the scientific literature, interventions offered through an app while people are engaged in their daily routines are known as ecological momentary interventions^14,15^, and when the intervention is adapted (e.g., the timing, intensity or type) over time to individuals’ changing circumstances in order to provide precision support, they are known as just-in-time adaptive interventions^16^ (JITAIs). In order to provide precision support, JITAIs execute decision rules at each pre-specified decision point, i.e. time points in everyday life when interventions can be delivered. These decision rules take dynamic information about an individual as input and link it to the most suitable intervention option. Just-in-time adaptive m-Health interventions have two novel characteristics. First, they do not require individuals to know when an intervention would be most useful or remember to access an intervention. These interventions are initiated through a push mechanism (SMS, notifications, etc.). Second, what the app offers, and when, can change according to the temporal dynamics of individuals’ everyday circumstances. Such interventions are dynamically initiated by the app at a time determined to be beneficial. These types of m-Health interventions have been explored in smoking cessation^17^, increasing physical activity^18,19^ and enacting cognitive tasks like self-reflection^20,21^. By initiating support in this adaptive manner, at times when individuals are likely to benefit in their environment, m-Health resources developed for mental health care can substantially improve engagement and outcomes.

There are two components that must be learnt to provide precision support while people are engaged in their daily routines. First, it is necessary to identify whether providing an intervention at pre-specified time points is likely to be most beneficial to an individual. Such time points are hypothesised as when an individual is able to practice the suggested behavioural action in daily life and benefit from it. But individuals will act only if they are sufficiently motivated, able and do not find it burdensome to practice the suggested action at these time points. For example, if an individual is currently operating a vehicle, then the individual is unable to follow suggestions to perform mood uplifting physical activity behaviours. Second, it is necessary to identify the best intervention option for the state an individual is in at the moment the intervention can be offered. To better understand, consider an anxiety intervention at a time an individual is having negative thoughts. In this state, a nudge to ‘practice slow breathing techniques’ is likely more useful than an activity to reflect on the pros and cons of trigger thoughts. Environmental and social circumstances of an individual at the time points interventions can be offered are just as important to help guide the selection of intervention. For example, if it is currently raining, then it may be more effective to suggest an indoor mood uplifting physical activity behaviour as opposed to an outdoor mood uplifting physical activity behaviour.

The data collected from traditional between-person randomised trial designs, such as randomized control trials (RCTs), is best suited for evaluating an already constructed JITAI. RCTs are not designed to inform the construction of a JITAI, that is, the personalised, optimal sequence of intervention options that apps should offer to an individual over time. New types of optimisation trial designs are needed to enhance m-Health apps to initiate interventions with precision—that is, provide the right intervention option at moments when it is most likely to be effective for the individual. This paper outlines the micro-randomised trial (MRT), a longitudinal experimental design to optimise the use of m-Health resources deployed in mental health care. The paper also illustrates this approach in a case study. It further reviews the design and implementation issues that need to be considered while conducting an MRT. The paper serves as a practical guide on MRTs to m-Health researchers in psychiatry with a checklist of common tips and pitfalls to avoid (Table 1).

**Table 1:** Checklist.

Issues	Considerations
Choice of distal and proximal outcome measure	What distal health outcome is being targeted? What is a suitable proximal outcome and how does it relate to the distal outcome? Is the proximal outcome measurable? Is the proximal outcome likely to change in response to the intervention used? What time duration should we use to derive the proximal outcome? Are sufficient engagement strategies in place to obtain reliable and valid proximal outcome measures?
Intervention options	Which intervention options might be actionable if delivered via mobile device in everyday life? Is the timeliness of content of the intervention option critical? How should the intervention options be delivered? By which mediating variables do you think the intervention option will impact the long-term health outcome? How should this intervention option impact the mediating variables in the near-term? Can you observe/record the near-term impact of this intervention option? How might temporal characteristics of an individual’s psychosocial, behavioural, psychological, or symptomatology factors influence the relative effect of the intervention option? Over what time interval do you think the intervention option will have the largest effect?
Choosing intervention delivery decision points	When is the user at increased risk? When is the user likely to be most receptive/responsive? Are there set times at which the user is most likely receptive/responsive or most likely not receptive/responsive? What means are there to detect in-the-moment receptivity? Can these detections be done in real time? Are there any fixed times at which the user might not be available? Can you detect in-the-moment unavailability? Are data collection and monitoring strategies reliable enough to detect decision points?
Randomising when and what	Determine how much burden a user can tolerate. Decrease probability of randomisation with increased burden and less tailoring
Ethical considerations	Give users control to decide when they do not want to receive interventions In some populations, there should be expert clinically determined cut off points regarding symptom severity that will trigger direct clinical contact. Consider domain science, ethical and self-determination rationales in designing intervention options Do you have m-Health & biostatistics skillsets? Do you have someone in the team who can guide the app developer to gather useful data for analysis? Are you recording what data is missing, when and why?

## Micro-randomised trial

### Overview

The micro-randomised trial (MRT) is an experimental design useful for constructing the decision rules for providing precision support in a JITAI m-Health intervention^22^. In this trial design, individuals are randomised at each decision point to an intervention option (i.e., whether to intervene and which intervention option to deliver based on the individual’s time-varying state). Consider a reminder to take one’s daily medication. Here the decision points would be daily and thus each participant might be randomised over 100 times in a 4-month study. In an MRT, at each randomised decision a proximal outcome measure is obtained (e.g.: mood over the next hour or daily step count). A sequence of analysis can be carried out using the MRT data to collectively build up evidence for the formulation of decision rules used to personalise the delivery of intervention options—what and when—with precision. The primary analysis is concerned with the marginal effect, that is the average over time, of the contrast between the two possible intervention options. In secondary analysis, moderation with the goal of understanding in which circumstances one intervention option is more effect, can be explored.

### Case study

We illustrate the utility of MRTs through a recently completed study that used an app-based intervention to enhance well-being in the US office worker population. The intervention component is a tailored push-notification prompt delivered in daily life as individuals are immersed in chores and daily schedules. The team did not want to overburden workers by sending too many prompts. Thus the intervention options were to either send the push-notification prompt or not to send a prompt. The message content of the push notification is tailored to the behavioural and emotional state of the recipient (assessed from sensor and self-report data)^23^. The near-term aim of initiating a prompt at a particular time is to persuade the participant to open the app and practice a self-reflection exercise very soon after receiving the prompt. The brief exercise involves a short self-reflection followed by reporting values—on a scale of 0 (worst) to 100 (best)—for daily energy, willpower, sleep, presence, physical activity, creativity, eating and perceived alignment with the community, work and personal purposes. The long-term objective is to habituate this self-reflection behaviour into daily routines in order to foster and maintain health and well-being through repeated practice. Prompting participants to engage in self-reflection exercises may also encourage them to self-monitor and be more aware of both symptoms and side effects they experience over time in their natural environment. As self-monitoring is a central paradigm in mental health care and research, even a modest improvement in knowledge on how a prompt intervention persuades participants to engage in an intervention can contribute to improved outcomes in mental illnesses.

**Fig. 1:**
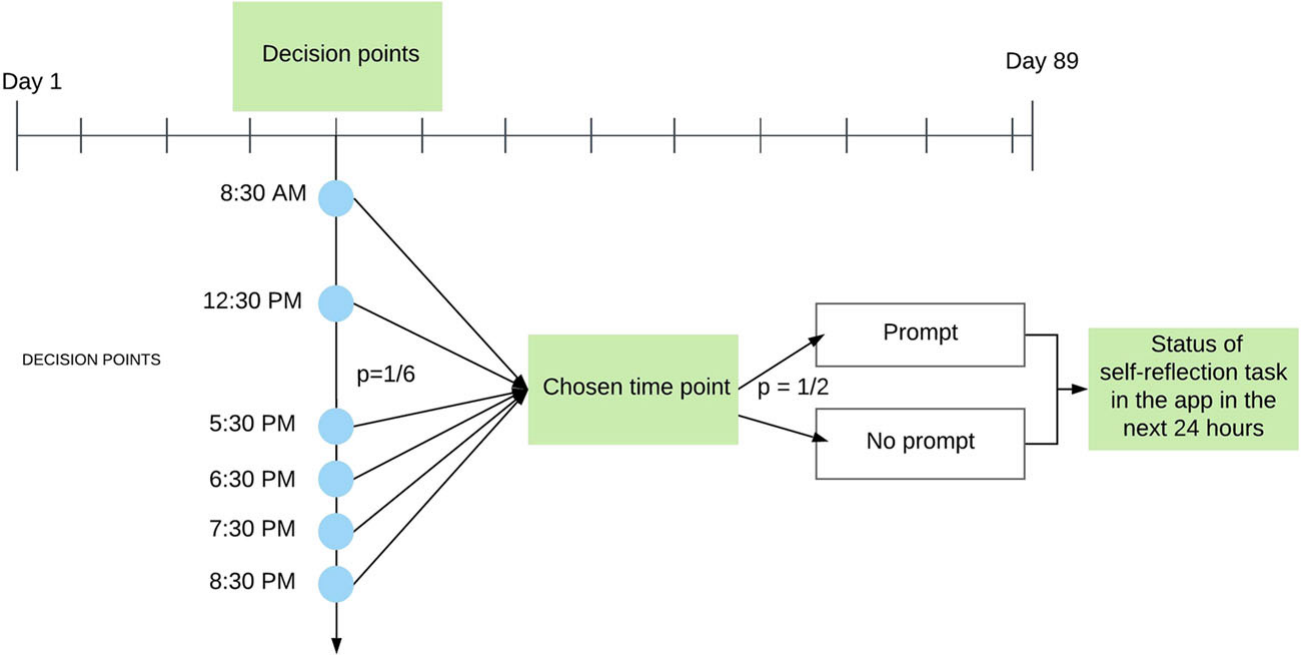
Flow of a micro-randomised trial design case study design. There were 534 intervention delivery decision points that consisted of six times distributed across the day over 89 days. The decision to choose a daily time and prompt intervention delivery at the chosen time were randomised. Engagement with the intervention in the app within next 24 hours was the proximal outcome measure.

Only when a prompt is sent at an appropriate time is it likely to succeed at persuading a participant to complete the suggested self-reflection exercise. An appropriate time is defined as when the participant will see the notification, and have sufficient time, energy and motivation to pay attention to the intervention message and perform the reflection activity in the app. As this app was used by office workers in the US, it was hypothesised that the best time to do a self-reflection exercise was during free time. Prior research suggests that less busy times for office workers are in the morning, during a lunch break and in the evening after work. An MRT was implemented to learn first, whether a prompt initiated at six time points (Fig. 1) is effective, and second, how the effects of push notification varied over time. The trial protocol involved first choosing a decision point in a day with one-sixth probability. The choice of six decision points spread the distribution of convenient times throughout the day. At each chosen decision point, participants were randomized with 50% probability to either receive or not receive a push notification containing a tailored health message. This repeated randomisation over time accounts equally for the effects of unobserved biases from temporal confounders, thus both the causal effect of the intervention options as well as the causal relationship between time and intervention options’ effects can be examined. The trial was conducted with 1255 eligible app users over 89 days with 534 decision points (six times per day over 89 days).

## MRT implementation considerations

### What is an appropriate proximal outcome?

The proximal outcome is a measurable near-term effect observed after receiving a m-Health intervention in everyday life. It is usually a mediator of a validated and well-established longer term health outcome that is of clinical importance (e.g., remission from depression at 6 months). A behaviour or self-efficacy metric collected frequently could be chosen as proximal outcome measure. For example, daily physical activity, smoking abstinence days, daily sleep variability can be near-time indicators in mental health interventions, as these are important mediators of self-efficacy and recovery. With chronic mental illnesses, health process measures such as adherence to medications and clinic appointments—where there is ample evidence that patients who consistently enact these processes maximise their recovery—can be suitable proximal outcome measures. These measures could be obtained through the app, or by external means. While choosing the proximal outcome, it is crucial to that the impact of the targeted near-term outcome on long-term health outcomes is well established. It is also important to assess near-term outcome measures at appropriate time periods. After an intervention has been delivered, outcomes should be assessed within the shortest time period where change is anticipated.

### What type of interventions are appropriate?

An appropriate intervention for an MRT can be initiated during the course of everyday life, must be actionable within a short period of time, and should lead to a likely benefit on a near-term basis. Interventions of frequent support to manage daily or weekly issues/risks that arise frequently fall under this category. For example, an individual in recovery from alcohol use disorder may be assisted by an intervention recommending them to connect with a peer support mentor on high relapse risk days. Outside the high-risk days, an intervention in the form of a suggestion designed to increase suitable behavioural repertoires or cognitive reframing strategies to better understand what causes relapse may also be suitable. MRT interventions can also focus on influencing in-the-moment choices. For example, providing suggestions for shopping ideas that are consistent with diabetes management goals when the app detects the individual is located in a grocery shop. However, not all brief interventions are suitable for a MRT. If an intervention can only be provided when a high-risk event occurs (e.g. at times when suicide attempts are likely), and these risk events are rare or infrequent, then a MRT is not suited for this intervention, unless there are a vast number of subjects. While suicide attempts or self-harm interventions are not suited due to the fact these are rare events, interventions targeting suicidal ideation or thoughts are suitable for MRTs when these events are likely to occur frequently among the targeted population.

It is equally important to select interventions that are acceptable to the target population, such as behaviours participants find motivating and are able to action frequently in their setting. Through the course of everyday life, participants might be able to perform the behaviour specified by the intervention across a range of time points. A number of these time points can be selected for intervention delivery using principles outlined in the next section. Lastly, interventions must be suitable for delivery in an m-Health format^24^. As a push notification in the app can be programmed to lapse after a set time, intervention options sent through such a notification can be designed to be actionable within a very short amount of time; intervention options that are sent through text message should not be designed with a short expiry time as there is no way to control how long they are visible on the phone once sent.

### How to choose intervention delivery decision points?

Decision points are all the possible times during an individual’s daily schedule that it may be feasible to deliver an intervention option. The more frequent the decision points are in time, the more real time the interventions appears to individuals. However, to enhance the ability to detect effects, it is often important to narrow the time points at which intervention decisions will be made. Choosing appropriate decision points requires an understanding of the temporal resolution at which an intervention should be offered, and how quickly the near-term impact of the intervention option can be observed. These are domain-specific factors. It might be plausible to use past m-Health studies to identify and eliminate time points that have shown limited or no outcome when interventions were offered. Equally, domain knowledge can also be useful for choosing decision points, as it can help narrow down the times where there is some evidence the intervention will have a near-term impact.

When interventions target similar populations, prior knowledge can be useful to localise times for decision points. In such a situation, either everyone does similar things, or their lives are structured in a similar manner, and times for decision points can be constrained. For example, in a workplace population, most probable decision points in a day to practice a stress related relaxation technique can be narrowed down to before work, during the lunch break, and after work, as these are the times at which people are more likely to have time and energy to pay attention^9^. Even though the encoding of these decision points is localised at a domain level, they can in fact be actioned in a person-specific manner. For example, “before work” or “around lunch” can occur at different times for different individuals but have the same meaning for all. Users can be asked to pre-specify times that correspond to these decision points for their situation^10^.

On the other hand, the best times to offer an intervention option for inhibiting a craving or overcoming an anxious thought (best offered in moments of crisis) are not similar across individuals, and the decision point times have to be selected at an individual level. In this scenario, since we might not have prior knowledge of when these moments might be, or the knowledge we have suggests substantial variance from person to person, choosing as many decision points over time as possible would be the best course of action. For example, the decision points for interventions that are best practiced during a panic attack are best chosen at a minute resolution, as panic attacks can arise spontaneously.

The choice of decision points in an MRT is critical as the interventions may only be potentially delivered at those time points. This constraint limits learning if individuals are either not receptive, or not in the required state at the chosen time points.

### Randomising: when and what?

Several papers elaborate in detail how to calculate power and sample size for an MRT^22,25,26^. The objectives of the MRT, coupled with sample size, should inform which intervention options should be randomised at which time points. In addition, the current state of the individual restricts which intervention options are appropriate. For example, if the individual’s context is ‘driving a vehicle’, the only usable intervention option is ‘not to interrupt’. Alternatively, if the current context is ‘walking’, intervention options that suggest ‘to extend current walking activity’ may be suitable for experimenting. We should only randomise at time points when it is possible to intervene with contextually appropriate intervention options.

Furthermore, how much burden individuals are willing to tolerate and when should be considered while determining the probability of randomisation. Intervention options that pose a high burden on individuals should be randomised to be offered less frequently over time. For example, a 60-min intervention activity might be more appropriate when offered once every 2–3 days, while a brief 2-min activity could be offered a few times each day. Equally, if a particular intervention type provides the same or similar content at all time points and contexts, there is a risk of habituation. Intervention options that are less tailored to the individual should also be randomised at a lower rate to reduce burden and habituation, as there is ample evidence to suggest that greater personalisation increases engagement^26–28^.

### Ethical issues

It is important to ensure appropriate mechanisms are in place that allow participants to provide informed consent to participate in the MRT. Since an intervention delivered in as an individual goes about their life could be intrusive, it is important not to infringe on individuals’ rights to self-determination. Individuals must be receptive to receiving an intervention at the time it is delivered on their own accord. Options that allow individuals to control when and what type of interventions they will not receive should be incorporated in the MRT. For example, the app settings could include an opt-out option that allows individuals to turn off notifications for a set period of time. For some study populations, for example, people with severe depression or suicidal ideation, expert clinicians should advise on cut off points regarding the symptom severity which initiate direct clinical contact with the subject.

### How to ensure that an implemented MRT results in actionable data?

Documentation of missing data is integral to ensure the MRT implementation results in useful and actionable data. Unlike conventional trial data, data in an MRT are being collected and used through a smartphone app in real time to decide when to intervene, the content of the interventions and to measure the immediate near-time intervention response. For example, users’ current stress level (assessed from sensors or self-report) could determine when and what type of intervention options are randomised. If some aspects of the information necessary to estimate stress are not captured at a particular decision point, then certain intervention options may be excluded at that point. The nature of the “missingness” in the real-time information influences decisions of when and which type of intervention options are being experimented with. Not factoring the effects of “missingness” into the analysis can bias the outcomes. To make the generated data actionable, the app developer should be instructed to record—to the extent possible—when intervention options were not delivered at times they were intended. Why they were not delivered should also be recorded: was the user out of wireless range, for example, or was there a software glitch? In addition, when an individual’s context is unavailable and why it was not available should be recorded. Since individuals update their phone software sporadically, recording the version of the app and the version of the operating system used at the time of each decision point can help explain data problems caused by upgrades^29^. It is also imperative for the research team to brainstorm which context variables might moderate the effect of the intervention option on the near-term outcome and ensure that these variables are also recorded.

Getting the MRT implemented within the constraints of technology, budget and time poses several questions including: should the functionality of an existing app be expanded or a new app developed, and does randomisation occur locally within the app or in the cloud. It is important to ensure the study team includes someone with an m-Health skillset who can serve as an intermediary between domain experts, app developers and biostatisticians.

## Discussion

Despite the potential benefits in mental health care delivery, low uptake and use of m-Health resources in the real world suggest engagement issues. A challenge of m-Health resources is that their interactions with individuals can be generic, and therefore impersonal. Personalisation to initiate interventions precisely at moments when they are most likely to be effective for the individual is one possible way to address this problem. The MRT is an experimental approach to personalise m-Health resources. m-Health resources implemented with an MRT initiate interventions at all possible times in a randomised manner, and at the moment of intervention if several suitable options exist, one is chosen at random. The data generated from an MRT can be used to make causal inferences about the effects of intervention timing and content on a near-term outcome, which can be used to set when and what an app should initiate with precision. m-Health interventions can thus be integrated into daily routines. Our checklists cover key issues pertinent to designing and executing a micro-randomised trial with m-Health interventions that are not encountered in traditional experimental approaches.

In a micro-randomised trial, everything happens on the run and in an online environment that makes it critical to have appropriate technical expertise. These trials both collect and make use of data in real time to determine the content of the intervention options, to randomise, intervene and measure outcomes. It is essential to make sure that interventions are sound both scientifically and ethically. These types of interventions differ from traditional internet interventions (made available through apps and websites) which individuals access at their will, when they think it is helpful. In an MRT, each participant experiences a unique, dynamic intervention that changes over time. Participants could be at risk of interruption at inappropriate times as well as lose the ability to practice self-determination due to the nature of these interventions. Expertise in user-centred and participatory design approaches are required in designing an MRT to mitigate these possible effects on participants. Biostatistics skills are also necessary to accurately analyse MRT data to ensure valid causal inferences and appropriately adjust of correlations in longitudinal outcomes over time. These inferences can include assessments of whether interventions delivered at one time point might have a carry-over effect on the proximal outcomes of interventions at later decision time points. Furthermore, prior intervention may moderate the effect of current intervention options on proximal outcomes.

An area that we have not covered is methods for randomising among intervention options at the decision points in MRTs. Unlike conventional trials, in an MRT, each individual needs to be repeatedly randomised to intervention options at various decision points during the course of the trial. Furthermore, not everyone will have decision points at the same time. Participants might need to be randomised several times a day, with a series of intervention options and times unique to them. In some instances, the state of the user in the moment determines if that an intervention should be delivered at that decision point or not. One approach is to use machine learning algorithms to randomise time of delivery or intervention option or both online and in real time^30^. The algorithms themselves can be implemented in the app, or remotely run on a cloud server communicating with the app. Multiple intervention components can be investigated in parallel each with different intervention options^31^. This approach is particularly useful to learn simultaneously how to different intervention components might either positively or negatively interact. Other relevant details include sample size calculation methods^11,13^ and statistical analysis techniques to negate the cumulative effects of previous time point outcomes on current ones^20^. Issues crucial to the collection and management of MRT data along with steps to identify sources of missingness have been discussed in detail by Seewald et al.^32^.

Lastly, it is crucial to mitigate a common data collection pitfall that arises in m-Health research because of using the same strategy to gather outcome measures used for both intervention and evaluation purposes. In an m-Health intervention outcome measures might be used to tailor intervention content, or to decide whether or not to provide an intervention. Outcome measures are also used to evaluate intervention response, such as how useful the app is and how much it is being used. Research studies use incentives as a strategy to compensate participants for providing outcome measures needed for evaluation. But incentives are not part of the intervention. Hence, it is important to ensure that individuals are not being paid for providing outcome measures used in evaluation, if the same outcomes are also being used to tailor interventions.

## Conclusions

A unique advantage of m-Health resources in mental health care delivery is the capability to deliver personalised interventions precisely at moments of need, and thus help individuals to enact behaviours in an easy-to-scale and low-cost manner. Traditional m-Health resources are designed using a *one-size-fits-all* approach: what is offered and when does not vary with the temporal dynamics of factors affecting individuals’ daily lives. The micro-randomised trial (MRT) is a new experimental approach to personalise m-Health interventions. Employing this approach provides data needed to understand when it is most useful to initiate an intervention through the app, and which intervention options are best suited to the circumstances of the individual at particular moments. The checklist below outlines several steps and suggestions in important areas of micro-randomised trial design and execution. We hope this checklist will help m-Health scientists implement successful MRTs that yield useful data.
